# Investigating the potential of Zernike polynomials to characterise spatial distribution of macular pigment

**DOI:** 10.1371/journal.pone.0217265

**Published:** 2019-05-24

**Authors:** Piers Allen, Antonio Calcagni, Anthony G. Robson, Ela Claridge

**Affiliations:** 1 School of Computer Science, University of Birmingham, Birmingham, United Kingdom; 2 Aston University, Ophthalmic Research Group, School of Life and Health Sciences, Aston Triangle, Birmingham, United Kingdom; 3 Moorfields Eye Hospital NHS Foundation Trust, Department of Electrophysiology, London, United Kingdom; 4 Institute of Ophthalmology, University College London, London, United Kingdom; Justus Liebig Universitat Giessen, GERMANY

## Abstract

It has been postulated that particular patterns of macular pigment (MP) distribution may be associated with the risk for eye diseases such as age-related macular degeneration (AMD). This work investigates the potential of Zernike polynomials (ZP) to characterise the level and distribution of MP, and their suitability as a representation for analysis of the effects of age and AMD on MP patterns. As the case study, MP distribution maps computed using an experimental method based on fundus reflectance (MRIA) were obtained for ninety volunteers representing three groups: under-fifty without AMD, fifty and over without AMD, and fifty and over with AMD. ZP with 105 coefficients were fitted to the maps using least-squares optimisation and found to represent MP maps accurately (RMSE<10^−1^). One-way MANOVA analysis carried out on ZP representations showed that the three subject groups have significantly different means (Wilk’s Lambda 0.125, p<0.0001). Linear discriminant analysis with leave-one-out scheme resulted in accuracy, sensitivity and specificity of classification according to, respectively, disease status regardless of age (81% all); disease status in the age-matched groups (87%, 88%, 86%); age irrespective of disease status (81%, 83%, 73%); and age for subjects without AMD (83%, 88%, 80%). Mean MP distributions computed from ZP coefficients for the three groups showed more elevated and more peaked MP for the healthy under-fifty group; more irregular and more elevated peripheral levels in over-fifty AMD group than in over-fifty non-AMD group; and moderate radial asymmetry in non-AMD over-50 group. The results suggest that ZP coefficients are capable of accurately representing MP in a way that captures certain spatial patterns of its distribution. Using the ZP representation MP maps could be classified according to both age and disease status with accuracy significantly greater than chance, with peak elevation, pattern irregularity and radial asymmetry identified as important features.

## Introduction

Age-related Macular Degeneration (AMD) accounts for over 50% of all cases of registered blindness in people over 65 years in the UK [1]. It is a progressive disease affecting the macula, a small region in the centre of the retina responsible for detailed vision. The macula is a circular area of approximately 6mm in diameter, which may also be defined as the portion of the posterior retina that contains two or more layers of ganglion cells. It comprises a cone-dominated fovea, which measures approximately 1.5mm, with the cone-only foveola (approximately 0.35mm) at its centre and surrounded by the parafovea and the perifovea [2, 3].

AMD is a multifactorial disease the pathophysiology of which is yet to be fully understood. A growing number of studies investigate its many aspects, from molecular underpinnings to demographic and environmental factors. A growing number of diagnostic methods for assessing risk of sight threating complications are becoming available to clinicians, from genetic testing to new imaging and image analysis techniques. There is growing evidence that the carotenoids lutein and zeaxanthin present in the macula, and collectively known as the macular pigment (MP), play a protective role [4], and that low levels may be associated with a higher risk of developing AMD [4–7]. The availability of methods for accurate and objective assessment of MP levels and distribution within the macula would be an important step in the timely commencement of supplementation and the investigation of potential prevention strategies [8].

### MP and foveal morphology

Macular pigment varies in concentration along the x, y and z axis of the retina. In brief, in healthy retinas, MP attains its highest concentration at the centre of the fovea and declines to negligible levels at a radial distance of 1-2mm from the centre [8–10]. Spectroscopically it acts as a broadband filter absorbing light at the blue end of the visible spectrum, with maximum absorbance at 460 nm [11]. Spatial and density distribution of MP is intricately related to retinal anatomy. These complex relationships are beyond the scope of this paper, and the interested reader is referred to the seminal works by Snodderly et al. [10, 11].

### Existing MP characterisation methods

Different techniques have been adopted to measure MP non-invasively in vivo [8, 12]. The techniques vary in their approach, effectiveness, accessibility and objectivity. Among the clinical methods, the most commonly used is Heterochromatic Flicker Photometry (HFP). It is a subjective psychophysical method whereby the subject is required to minimise the luminance contrast between a pair of rapidly modulated chromatic stimuli, as determined by the percept of minimum flicker. By choosing two wavelengths that are absorbed minimally and maximally by MP, it is possible to compute MP optical density (MPOD) at any macular location relative to a non-pigmented area, typically at the fovea. In more advanced methods, multiple measurements at different locations are taken to generate a 2-dimensional MPOD spatial profile. An analogous method involves minimizing the perceived motion of a bichromatic grating [13].

A number of techniques for objective measurement of MP have been developed in research laboratories, but have not yet been used routinely in a clinical environment [14]. The most promising of these are autofluorescence (AF), including two-wavelength autofluorescence (2W-AF), quantitative autofluorescence (qAF) [15, 16], fundus reflectance [17, 18], fluorescence lifetime imaging ophthalmoscopy (FLIO) [19, 20] and Raman spectroscopy [21]. Most of these techniques are capable of showing 2-dimensional quantitative topographic distribution of MP rather than a single relative MPOD value or 1-dimensional MP profile. There is not as yet a consensus as to how to best interpret, assess and compare these 2-dimensional distributions. Visualisations are commonly used to develop general intuitions about MP distribution in various populations. Measures used for quantitative analysis include the peak (maximum) value, total amount (concentration x pathlength x area) integrated over the MP region, eccentricity of the peak, the mean and standard deviation of the radius, and the gradient of radial distribution of the pigment [9, 22–25].

These assessment methods have been used in studies investigating a number of pertinent questions, for instance: Could MP levels be increased via dietary supplementation? [22] Do MP levels vary naturally as a function of age or gender? [14, 26, 27] Are MP levels decreased in patients diagnosed with AMD? [22]. To date, these questions have yet to receive an unequivocal answer and the need for further developments is recognised [5, 8].

This paper proposes the use of an additional set of measures derived from coefficients of Zernike polynomials, originally devised to characterise wavefront aberrations in beam optics, but also commonly used for fitting irregular surfaces over a circular region [28]. These measures are designed to capture some of the global indicators (peak magnitude, volume) as well as indicators of the symmetry and irregularity of distribution. As the case study, designed to test whether ZPs are capable of capturing differences between different populations, this work set out to answer two questions:

1. Do MP levels and distribution, as characterised by a number of indices derived from Zernike polynomials, differ between individuals with no evidence of macular pathology and those diagnosed with AMD?
2. Do MP levels, as characterised by ZP derived indices, show age dependency?

## Materials and methods

### Subjects

Images were acquired from 90 volunteers, distributed equally between three subject groups as shown in **Table 1**. All participants were assessed by an experienced clinician (AC) and assigned to one of the three groups, based on the clinical classification system for age-related macular degeneration [29]. Exclusion criteria for this study were: significant media opacities (defined as inability to clearly visualize the fundus on slit lamp biomicroscopy with a 90 dioptre lens); significant refractive error (defined as a spherical equivalent of more than 6 dioptres), and/or known retinal or choroidal pathology other than AMD; inability to give informed, written consent.

**Table 1:** Demographics of the experimental groups.

Group	Age	Diagnosis	Number of subjects	Males / Females	Mean age [range]	Number of images [acquired/analysed]
1	under 50	without AMD	30	18 / 12	32 [10–49]	238/203
2	50 and over	without AMD	30	13/17	70 [50–83]	237/153
3	50 and over	with AMD	30	10 / 20	73 [53–91]	132/123

Subjects in group 2 did not have any visible drusen / pigmentary abnormalities suggestive of AMD; subjects in group 3 were diagnosed with AMD (early, intermediate or late).

Participants in groups 1 and 2 were recruited from the pool of volunteers at Aston University and the University of Birmingham; participants were healthy individuals who had previously agreed to be contacted for research purposes. Participants in group 3 were recruited from the cohort of patients attending a local medical retina clinic who voluntarily contacted the research coordinators with a view to taking part in research studies on AMD. All applicable institutional and governmental regulations concerning the ethical use of human volunteers were followed during this research and the study protocol adhered to the tenets of the Declaration of Helsinki. Ethical approval for patient recruitment and image acquisition was obtained from the Research Ethics Committee at Aston University, Birmingham, UK, and all participants gave signed informed consent.

### Image acquisition

Images were acquired using a modified fundus camera (Zeiss RCM250) in which the original light source was replaced with a bespoke multispectral illumination system consisting of a white light source (OSL1 with 150W, 3250K halogen bulb; Thorlabs Inc., Newton, N.J., U.S.A.) and a computer-controlled tuneable filter (VariSpec CRI, U.S.A.). Six narrow-band filters (507 nm, 525 nm, 552 nm, 585 nm, 596 nm, and 611 nm; 7 nm full width at half maximum) were chosen by optimisation to simultaneously minimise the error of MP quantification and the acquisition time [30]. During the imaging session, three sets of images at the six specified wavelengths were taken in quick succession and captured by a Hamamatsu ImageEM C1300-13 cooled EM- CCD camera. The average acquisition time per image was 28ms [31]. The best of the three images at each wavelength was chosen for computing MP maps using MRIA. Only images of satisfactory quality were used for analysis. Those that were blurred, poorly exposed, or showed excessive displacement between frames, were excluded. MP maps were computed for regions of interest with eccentricity of 4 degrees, manually centred on the foveola.

### Multispectral retinal image analysis

The input to the analysis were topographic maps of macular pigment distribution computed using the multispectral retinal image analysis (MRIA) technique [32]. MRIA is based on fundus reflectance, and in common with some reflectometry approaches it explicitly exploits the relationship between spectral measurements and retinal architecture [17, 18, 33, 34]. The technique has been extensively described elsewhere [30, 32, 35–37]. It is briefly outlined below to provide context for the Zernike-based characterisation of MP which is the main topic of this study.

The MRIA technique is based on a predictive (forward) model of image formation which simulates photon propagation through the fundus tissues to establish a link between tissue composition and fundus appearance at different spectral wavebands. For each plausible combination of pigments occurring in the fundus (MP, retinal haemoglobins, RPE melanin, choroidal haemoglobins and choroidal melanin) a reflectance spectrum is computed using Monte Carlo simulation [38]. A collection of the predicted spectra forms the *reflectance model* in which each spectrum corresponds to one, and only one, combination of concentrations of the five above pigments [30, 32, 39]. Pixel-wise concentration of each of the pigments is then computed from multispectral reflectance images through *model inversion*. This can be done in a variety of ways [30, 37]. The inversion method used in this paper is based on Gaussian Process Emulation [40, 41]. One of its merits is that it can take into account the general characteristics of spatial distribution of the relevant pigments. In particular, in the macular region the MP is assumed to decrease monotonically from the central peak; retinal haemoglobins are assumed to be negligible in the foveal avascular zone, and then are monotonically increasing; RPE melanin is assumed to have elevated levels at the fovea, decreasing monotonically with eccentricity; choroidal haemoglobins and choroidal melanin have smooth distributions throughout the fundus [42–46].

**Fig 1:**
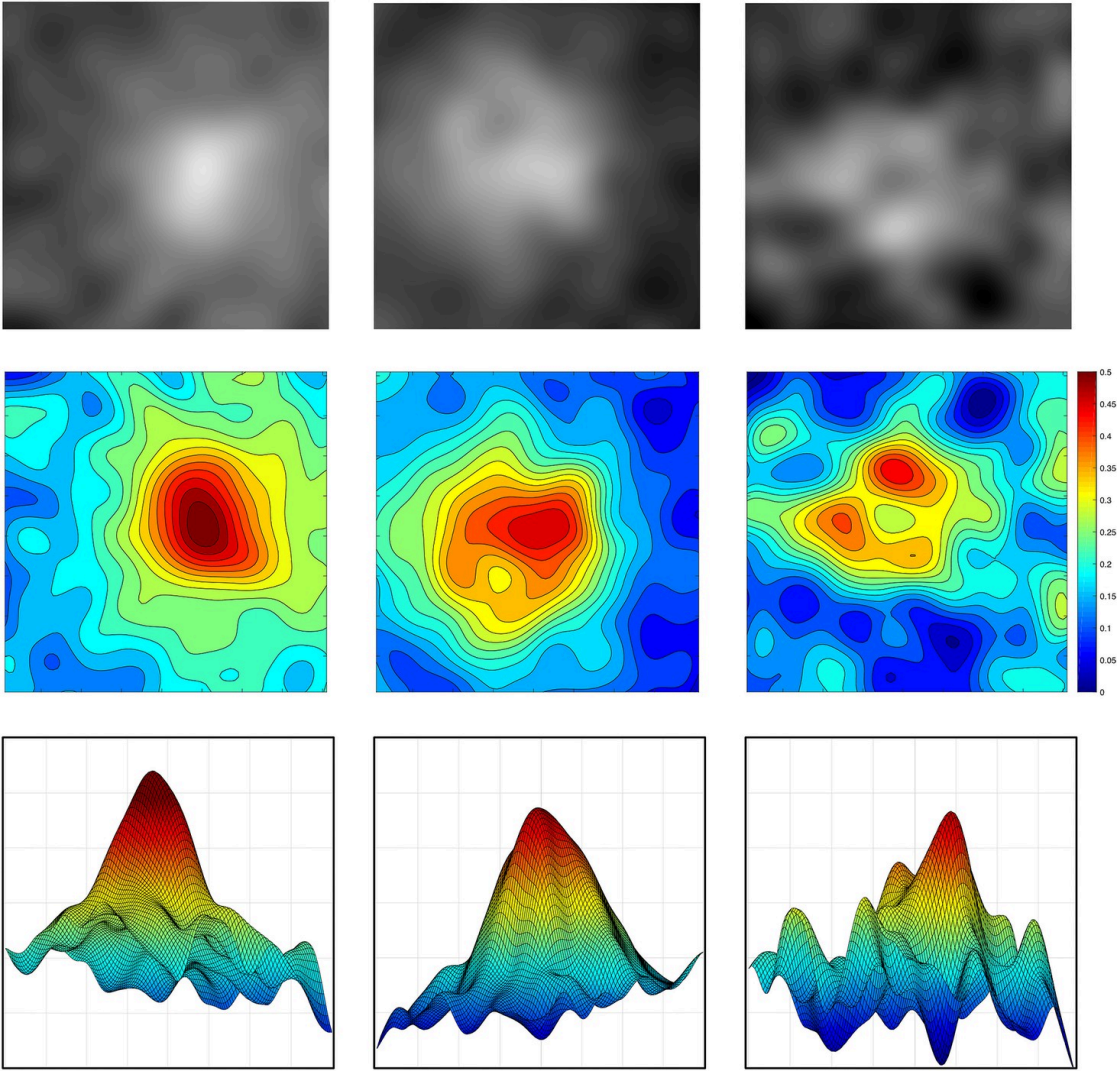
**Examples of MRIA maps** showing the magnitude and distribution of the macular pigment in subjects from group 1 (left), group 2 (centre) and group 3 (right). Top row: original MRIA maps of MP with brightness proportional to MP level; middle row: visualisation of MP as contour maps, derived from the original MRIA maps; bottom row: visualisation of MP as 3D elevation maps. The bar on the right shows the MP concentration (A.U.). The maps represent a region with eccentricity of 4 degrees centred on the fovea. All the MP maps are publicly available at the University of Birmingham eData Repository, “Macular pigment maps and groups”, https://edata.bham.ac.uk/153/.

A possible downside of the Gaussian Process Emulation is that the MP maps it generates do not have the sharp central peak typically seen with other imaging techniques. The rounded peaks seen in the MRIA maps are smoothed (spatially averaged) versions of the underpinning MP distributions. The three properties of interest in this study—peak magnitude, symmetry and irregularity–are preserved, albeit with a lower spatial resolution than the original, potentially noisy, signal. The loss of a sharp peak may be only mildly detrimental for analysis, as it has been suggested (e.g. [22–24, 47]) that the overall magnitude and patterns of spatial distribution could be more accurate indicators of AMD risk than the magnitude of the central peak alone. Examples of MP maps computed with the MRIA technique are shown in **Fig 1**.

### Zernike polynomials–theory–forward model

The Zernike Polynomials (ZP) are defined as a set of polynomials orthogonal over a unit circle [48]

$$ZP(\rho ,\varphi )=\sum_{n,m}C_{n}^{m}Z_{n}^{m}(\rho ,\varphi )$$ (1)

where *C* denotes the Zernike coefficients (amplitudes), *Z* the polynomial basis functions, ${|Z}_{n}^{m}|\leq 1,$ *ρ* is the radial distance, 0 ≤ *ρ* ≤ 1, and *φ* is the azimuthal angle.

There are two types of basis functions, even and odd. $Z_{n}^{m}$ is even when *m* is positive, and odd when *m* is negative:

$$\begin{array}{c} \mathrm{Even}:\;Z_{n}^{m}(\rho ,\varphi )=R_{n}^{m}(\rho )\;\cos\left(m,\varphi \right)\\ \mathrm{Odd}:\;Z_{n}^{-m}(\rho ,\varphi )=R_{n}^{m}(\rho )\;\sin\left(m,\varphi \right) \end{array}$$ (2)

The *n* and *m* are known as radial and azimuthal frequency indices respectively. The radial polynomial $R_{n}^{m}$ is defined as follows:

$$R_{n}^{m}(\rho )=\sum_{k=0}^{\frac{n-m}{2}}\frac{{\left(-1\right)}^{k}(n-k)!}{k!(\frac{n+m}{2}-k)!(\frac{n-m}{2}-k)!}\rho^{n-2k}$$ (3)

For notational convenience the OSA/ANSI sequential indices [49] are used in reference to the polynomial basis functions, defined as

$$j=\frac{n(n+2)+m}{2}$$ (4)

thus

$$ZP=\sum_{j}C_{j}Z_{j}$$ (5)

**Fig 2:**
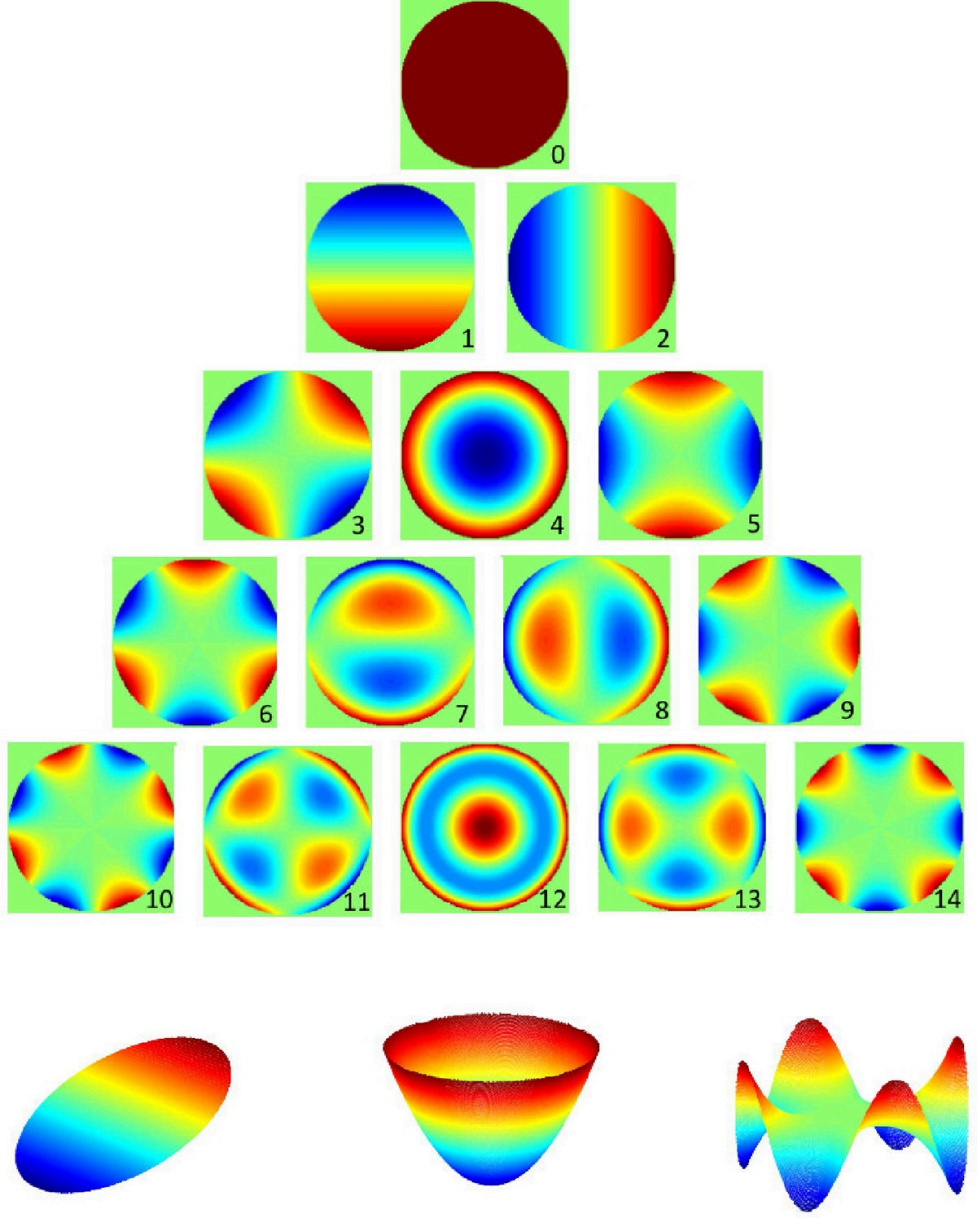
Visualisations of Zernike polynomials. Top: 2D visualisation of the first fifteen ZP on the unit circle. The numbers underneath the plots are ANSI sequential indices, see Eq (4). Bottom: 3D visualisations of ZP with indices 1, 3 and 14, representative of asymmetry, magnitude and irregularity of the periphery.

The Eq (3) can be used to generate 2D distributions, examples of which are shown in **Fig 2** (top) for the first 15 basis functions. It can be seen that, for example, term *Z*_0_ is a constant term, terms *Z*_1_ and *Z*_2_ are tilt terms, term *Z*_3_ represents a “cup” shape and term *Z*_10_ a “Mexican hat” shape. Other terms represent aberrations of varying orders. As a further illustration, **Fig 2** (bottom) shows 3D visualisations of terms *Z*_1_, *Z*_3_ and *Z*_14_ [50].

### Fitting zernike polynomials to image data

The analysis proceeds in two stages. Zernike polynomials are first fitted to MP images. The coefficients of the polynomial basis functions of the best fitting ZP are then used for classification of MP distribution.

The following property of ZP makes them an attractive choice for representing MP distribution: “*Any sufficiently smooth real-valued phase field over the unit disk can be represented in terms of its Zernike coefficients (odd and even)*, *just as periodic functions find an orthogonal representation with the Fourier series*.” [28]. In analogy with the Fourier Transform, this representation is referred to as the Zernike Transform.

A pixel patch centred at the macula and covering the adjacent parafoveal region is mapped to the unit disk. The objective is to find coefficients of the best fitting Zernike polynomial such that

$${\mathrm{ZP}}^{*}={\mathrm{argmin}}_{j\in J}\;d(MP,ZP(C_{j},Z_{j}))$$ (6)

where *MP* represents the image data, *C*_*j*_ is a vector of coefficients associated with polynomials *Z*_*j*_, and *d* is a difference measure. The solution is arrived at by optimisation.

To choose a suitable optimisation method three algorithms were evaluated: Levenberg-Marquardt, Quasi-Newton and non-linear least squares [51]. The input data consisted of ZP computed from randomly generated coefficient vectors. Accuracy was evaluated by comparing the generated and the reconstructed data using normalised RMSE as a difference measure *d*. Robustness was evaluated by assessing the accuracy of reconstruction for the ZP fields to which 10% Gaussian noise was added. The uniqueness of reconstruction was evaluated by computing Hamming distance between the original and the recovered normalised coefficient vectors where two coefficients were deemed to be the same if they differed by no more than 0.01 (1%).

**Table 2** lists the results of evaluation for 10 trials. The Levenberg-Marquardt method did not perform as well as the other two. The least squares and the Quasi-Newton method did not differ in performance, but computation time of the least squares method was two orders of magnitude less than that of the Quasi-Newton and therefore it was used to fit ZP to MP data. The maximum number of coefficients was set at 105 as any further increase did not reduce the fitting error.

**Table 2:** Performance of the optimisation algorithms.

	Accuracy [mean RMSE]	Robustness [stdev RMSE]	Uniqueness [mean Hamming distance]
Least squares	8.82 x 10^−2^	2.53 x 10^−4^	0.0
Quasi-Newton	8.82 x 10^−2^	2.53 x 10^−4^	0.0
Levenberg-Marquardt	8.83 x 10^−2^	2.83 x 10^−4^	0.9

Accuracy was evaluated on the noise-free data. Robustness to noise was evaluated by repeating the experiment ten times on the same dataset with different 10% uniformly distributed noise.

### Classification

To explore the potential of ZPs, this work has focused on two questions of relevance to MP studies: whether MP distribution varies as a function of age, and whether it varies between subjects with and without diagnosed AMD. The answers were sought by classification of MP maps represented by ZP coefficients. As shown in the previous section, a smoothly varying image data *MP* mapped onto a unit disk can be approximately represented by a Zernike transform:

$$MP=\sum_{j=1}^{J}C_{j}Z_{j}+\epsilon$$ (7)

where *Z*_*j*_ are indices of the Zernike basis functions (**Fig 2** (top)) and *C*_*j*_ are the associated coefficients obtained by optimisation. As the error *ϵ* was shown to be small (**Table 2**), it can be assumed that for purposes of classification the image data *MP* can be represented by a vector of coefficients

$$\bar{C}=<C_{j}>,j=0\ldots J,J\leq 104$$ (8)

#### Classifier selection

Four different classifiers available in the Matlab Neural Network Toolbox were employed: K-Nearest-Neighbours (KNN) (average error and 3-prioritised variant), Support Vector Machines (SVM), Softmax Layer Neural Network (SMNN), and Pattern Recognition Feedforward Neural Network (PRNN) [52].

Each classifier has a number of tuneable parameters (hyperparameters) which need to be chosen so that the classifier can produce the best results. The hyperparameters were determined by optimisation using, in most cases, the exhaustive search. The best performing parameters were chosen on the basis of ROC (Receiver Operating Characteristic) analysis, by finding the operating point closest to the perfect classification point where both sensitivity and specificity are equal 1 [53]. As Zernike polynomials are not translation invariant, classification was carried out using two versions of the image data: original, with the unit disk manually centred at the foveola; and translated, where the unit disk was placed to be centred on the maximum MP value (a peak) in the foveal region. This was to take into account the fact that in some MRIA images the central peak was indistinct (see the last paragraph of “Multispectral Retinal Image Analysis” section) and hence harder to pinpoint accurately. **S1 Table** lists the parameter values that resulted in the best performing classifier and **S2 Table** shows the results of classification with the above parameters.

#### Classification experiments

Classification experiments tested the following four hypotheses:

*Hypothesis 1*: MP distribution is different for subjects with and without AMD irrespective of age. Data from subject groups 1&2 vs 3 (all without AMD vs with AMD).
*Hypothesis 2*: MP distribution is different for age-matched subjects with and without AMD. Data from subject groups 2 vs 3 (all aged 50 and over, without AMD vs with AMD).
*Hypothesis 3*: MP distribution is different for different age groups irrespective of the disease status. Data from subject groups 1 vs 2&3 (aged under 50 vs all aged 50 and over).
*Hypothesis 4*: MP distribution is different for different age groups in subjects without AMD: Data from subject groups 1 vs 2.

In total 479 images were used (see **Table 1**). All input images had the form of an 81 x 81 pixel matrix with values between 0 and 1 representing the macular pigment concentration. Classification was carried out on ZP vectors derived from the image data using non-linear least squares fitting and using the best performing classifier identified above (PRNN). To overcome the problem of a relatively small data set the classifier was run a hundred times with data randomly split into the training, validation and test subsets.

### Analysis of spatial distribution patterns

It has been noted previously that a single estimate of the MP level in the macula, typically the peak value, provides insufficient diagnostic information [24]. In particular, several studies have found patterns of spatial distribution of MP to be statistically different in AMD and non-AMD subjects [54, 55]. It was therefore interesting to investigate whether ZP coefficients are capable of capturing patterns characteristics of each of the three subject groups. A feature selection algorithm based on a two-way t-test was used to rank the ZP coefficients in order of their significance.

As the number of coefficients (105) and the number of samples in each of the classification tests (**Table 1**) was similar, the ranking was repeated using a leave-one-out method (LOO) for each data item in turn. The most significant coefficients for each of the tests were computed as the *mode* of all the repetitions. Where this resulted in multiple occurrences of the same coefficient at a different ranking order, the coefficient at the highest ranking position was kept.

To investigate the interesting question of the spatial characteristics of MP distribution, Zernike basis functions corresponding to the highest ranking coefficients were assigned to three categories: those that are predominantly related to magnitude, those related to circular asymmetry of distribution and those related to pattern irregularity.

As well as coefficient ranking, Linear Discriminant Analysis (LDA) classification using the LOO method was repeated to investigate the effect of using a larger training set that this method provides in comparison to PRNN classification.

## Results and discussion

### Classifier selection

**Fig 3:**
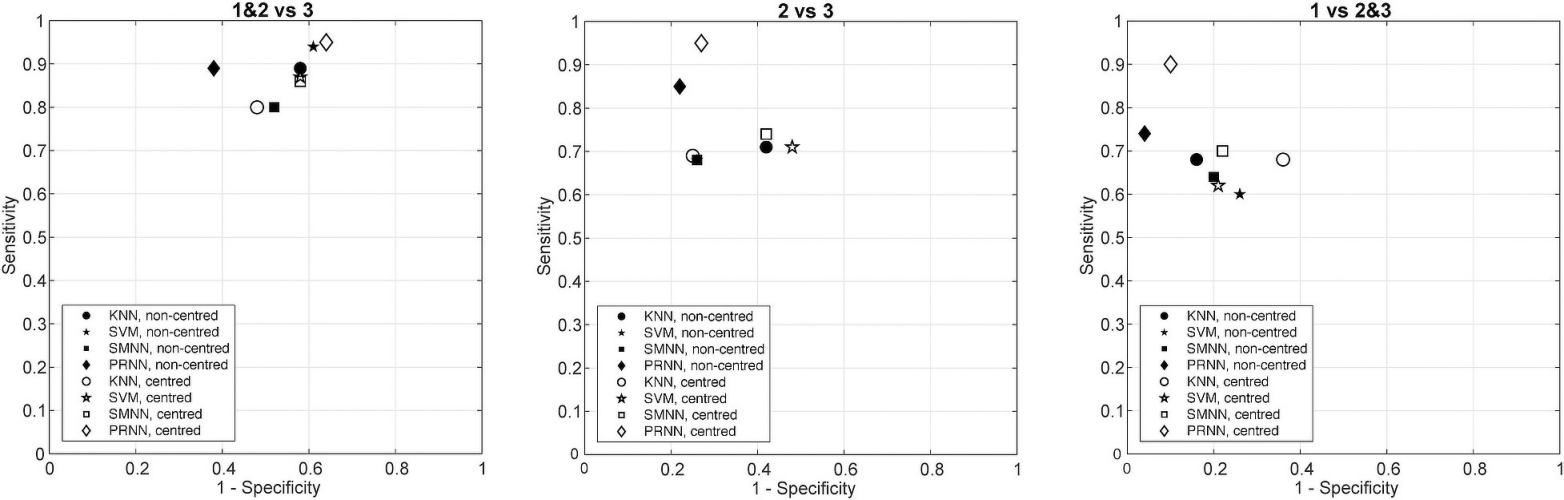
ROC plots of the results of the classifier selection.

The ROC plots visualising the results of the classifier selection are shown in **Fig 3**. The tabulated numerical results behind the plots are shown in **S2 Table**.

Classification accuracy varied significantly among the four methods investigated. The worst performing was KNN classifier, with SVM being only marginally better. The classification method that consistently produced best results was the PRNN with a high number of hidden neurons (see **S1 Table**), where the accuracy of most tests exceeded 80%. These findings suggest that Zernike coefficient representation of the MP data is capable of capturing some of the differential characteristics of the three classes, but the mapping between the coefficients and the classes is highly non-linear.

Considering the best performing classifier, PRNN, comparison of non-centred and centred data showed that the original (non-centred) data produced better classification across all the indices. This could be interpreted as an indication that the MP peak location, on- or off-centre of the foveola, is one of the factors contributing to the classification.

### Classification experiments

Results of the classification experiments related to the four hypotheses in the “Classification experiments” section are shown in **Table 3.**

**Table 3:** Classification using PRNN classifier with LOO selection.

Test groups	Mean			Best		
Not-centred	Accuracy	Sensitivity	Specificity	Accuracy	Sensitivity	Specificity
1&2 vs 3	0.74	0.77	0.43	0.85	0.84	1.00
2 vs 3	0.56	0.58	0.51	0.74	0.70	0.83
1 vs 2&3	0.68	0.62	0.71	0.78	0.94	0.73
1 vs 2	0.63	0.66	0.59	0.80	0.83	0.75
Centred						
1&2 vs 3	0.72	0.74	0.35	0.81	0.80	1.0
2 vs 3	0.54	0.56	0.49	0.67	0.65	1.0
1 vs 2&3	0.61	0.53	0.65	0.74	0.67	0.79
1 vs 2	0.61	0.65	0.56	0.74	0.70	0.82

Accuracy, sensitivity and specificity are shown according to disease status regardless of age (1&2 vs 3); the disease status in the age-matched groups (2 vs 3); age irrespective of disease status (1 vs 2&3); and age for subjects without AMD (1 vs 2). Columns on the left show the mean results for the LOO method, columns on the right show the best results among all the LOO tests. Test groups are described in Table 1.

Classification according to the disease status regardless of age (1&2 vs 3) resulted in mean accuracy of 74%, sensitivity 77% and specificity 43%; the best result among the hundred trials for this test had accuracy of 85%, sensitivity 84% and specificity 100%. It is likely that such a large difference between the mean and the best performance resulted from a well matched selection of the training and test sets in one of the random trials.

The mean results for classification according to the disease status in the age-matched groups (2 vs 3) were disappointing, with accuracy of 56%, sensitivity 58% and specificity 51%; the best results were significantly better, with accuracy of 74%, sensitivity 70% and specificity 83%. The combined number of datasets in groups 2 and 3 is relatively low in comparison to other tests which include group 1, the largest of the three, and this could have contributed to poor mean results. Classification was much improved in the LOO experiments (see below) where the number of samples in the training set were much larger (all but one sample in the whole set).

Classification according to age irrespective of the disease status (1 vs 2&3) resulted in mean accuracy of 68%, sensitivity 63% and specificity 71%; the highest scores in this test were accuracy 78%, sensitivity 94% and specificity 73%.

Classification according to age for subjects without AMD (1 vs 2) had similar mean accuracy of 63% and sensitivity of 66% but lower specificity of 59%; the highest scores for accuracy, sensitivity and specificity were 80%, 83% and 75% respectively.

Classification for the LOO experiments with LDA (**Table 4**) had results comparable to the best results using PRNN with either all 105 coefficients or a smaller subset of unique coefficients only. The main reason for a simpler method to achieve such a promising mean performance is likely to be a larger data set available for training in the LOO method. The results for centred data were marginally inferior to the results for non-centred data, similarly to PRNN classifier.

**Table 4:** Classification using LDA classifier with LOO selection on the ranked coefficients.

Test groups	All coefficients			N coefficients			
Not-centred	Accuracy	Sensitivity	Specificity	Accuracy	Sensitivity	Specificity	N
1&2 vs 3	0.81	0.81	0.81	0.80	0.78	0.80	95
2 vs 3	0.87	0.88	0.86	0.81	0.83	0.83	82
1 vs 2&3	0.81	0.83	0.78	0.81	0.81	0.78	93
1 vs 2	0.83	0.88	0.80	0.81	0.84	0.78	90
Centred	Accuracy	Sensitivity	Specificity	Accuracy	Sensitivity	Specificity	N
1&2 vs 3	0.77	0.76	0.78	0.79	0.73	0.81	98
2 vs 3	0.83	0.86	0.79	0.81	0.86	0.76	82
1 vs 2&3	0.80	0.83	0.76	0.80	0.82	0.76	96
1 vs 2	0.81	0.84	0.79	0.78	0.82	0.76	88

Accuracy, sensitivity and specificity of classification according to the disease status regardless of age (1&2 vs 3), disease status in the age-matched groups (2 vs 3); age irrespective of disease status (1 vs 2&3); and age for subjects without AMD (1 vs 2). Columns on the left show the mean results when using all 105 coefficients, columns on the right show the results when only N unique coefficients were identified by the classifier. Test groups are described in Table 1.

### Analysis of spatial distribution patterns

The experiments in which individual ZP coefficients were ranked according to their significance in classification produced very interesting results. **Table 5** shows the 15 most significant coefficients for each experiment. The entries are colour-coded according to the ZP type (magnitude, asymmetry and irregularity) with examples of each type illustrated in **Fig 2** (bottom). It can be observed that the highest ranking coefficients in the classification tasks involving subjects from group 1 (under 50, no AMD) against the other groups were mostly related to the magnitude of MP. This is in contrast to the classification according to the disease status in the age-matched groups (2 vs 3), where the highest ranking coefficients are related to irregularity of pigment distribution. Interestingly, in the classification according to the disease status regardless of age, there is a mixture of magnitude- and irregularity-related coefficients, with prevalence of the latter ones. Asymmetry related coefficients appear to matter mainly in distinguishing subjects aged under-50 and over without AMD where irregularity plays a lesser role.

**Table 5:** First 15 ZP coefficients ranked highest according to their significance using a feature selection algorithm based on a two-way t-test.

Test group	Fifteen highest ranking ZP coefficients for each test														
1&2 vs 3	4	14	12	76	55	101	78	91	48	25	95	58	36	71	64
2 vs 3	76	14	101	61	48	5	84	55	62	51	1	95	32	85	90
1 vs 2&3	4	12	55	14	78	91	7	66	64	92	67	49	36	25	1
1 vs 2	12	3	7	1	67	91	78	49	21	66	55	57	8	46	10

The numbers are ANSI sequential indices, see Eq (4). Colour coding indicates the principal pattern of a given ZP coefficient as follows: blue–magnitude; green–asymmetry; red–irregularity of the periphery. Test groups are described in Table 1.

Another interesting observation relates to indices of the ZP basis function (see section “Zernike polynomials”). Lower ones tend to occur more frequently in the tests involving under-50 non-AMD subjects (group 1), higher ones in over-50 AMD subjects (group 3). In the ZP indexing convention used in this paper, higher ZP numbers correspond to higher azimuthal frequencies. This suggests that in the age-matched groups the AMD and non-AMD subjects have different spatial variability of MP distribution. When comparing the actual coefficient magnitudes, those high-numbered basis functions have higher magnitudes for subjects from group 3 than those from group 2, suggesting irregular patterns with higher frequency for group 3, AMD subjects.

**Fig 4:**
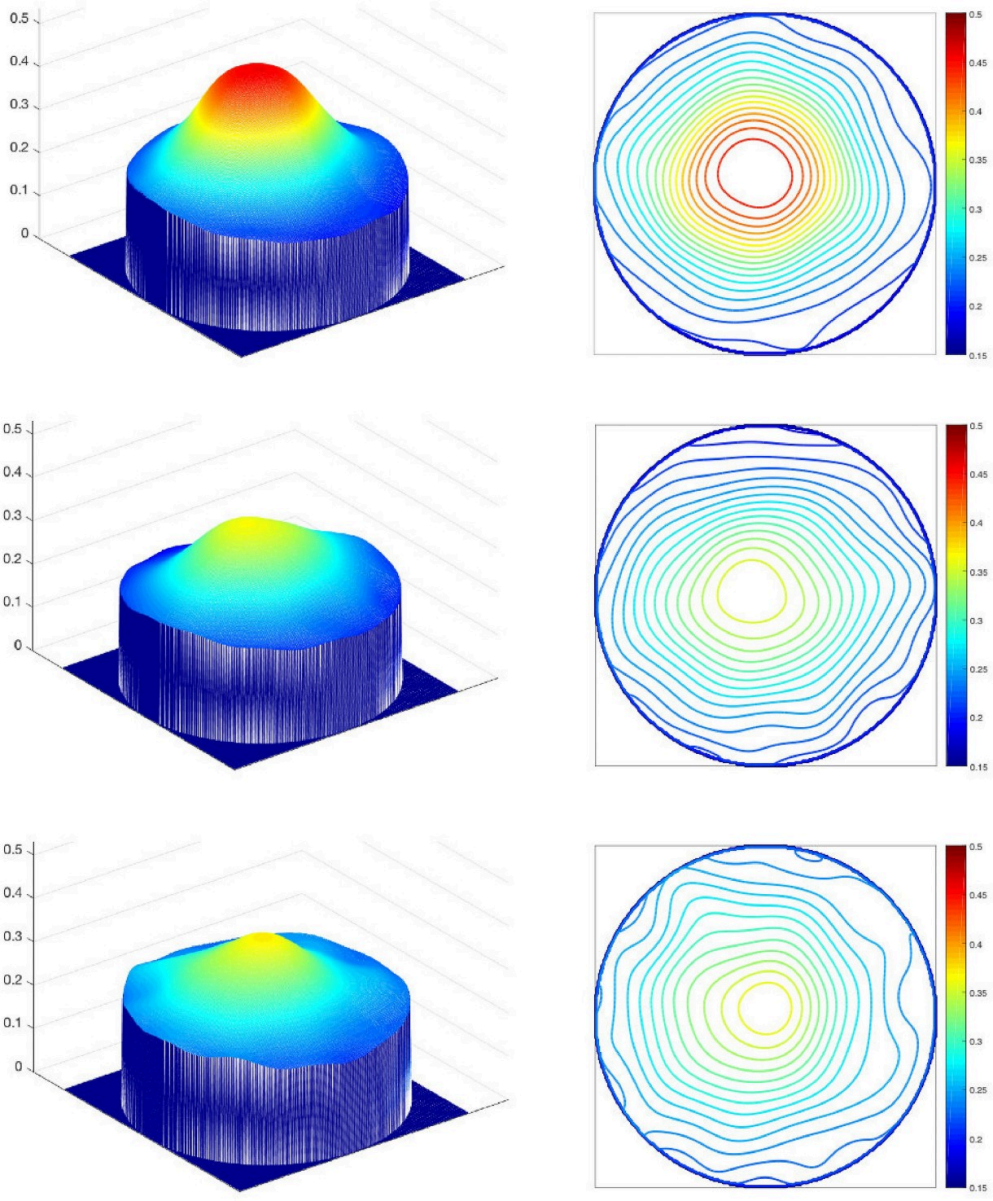
Mean MP distribution maps visualised as 3D elevations and 2D contours for three subject groups. (top row) subjects under 50 without AMD; (middle row) subjects 50 and over without AMD; (bottom row) subjects 50 and over with AMD. The maps were generated from Eq (5) using mean ZP coefficient values for each of the groups on the basis of classification results from the LOO experiments.

Finally, visualisation was used to see whether conclusions regarding the spatial distribution patterns are reflected in the actual appearance of the patterns representative of each class. Mean ZP coefficient values were computed for each of the three groups on the basis of classification results from the LOO experiments. MP distribution maps were constructed using Eq (5) with all 105 coefficients. They are presented in **Fig 4** as elevation maps and as contour maps.

Visual comparison confirms the numerical findings. MP for group 1 shows an elevated central magnitude in comparison to the other two groups. MP for group 2 differs from the other two groups by distribution which is not rotationally symmetric. MP for group 3 shows increased irregularity of peripheral MP distribution. One additional difference distinguishing group 3 from the other two groups is an increased level of MP at the periphery, or, in other words, a smaller relative elevation of MP at the centre. This is something that would be difficult to capture by the ZP representation as it is the result of a particular superposition of many basis functions.

## General discussion

This study investigated the potential of Zernike polynomials to characterise the level and distribution of MP by a set of abstract indices and the ability of this representation to examine associations of MP patterns with age and AMD.

Zernike polynomials with 105 coefficients were found to represent MP maps accurately (mean RMSE < 10^−1^, **Table 2**). MANOVA analysis carried out on ZP representations showed that the three subject groups (under‐fifty without AMD, fifty and over without AMD, and fifty and over with AMD) have significantly different means (Wilk’s Lambda 0.125, p<0.0005).

Subsequent classification experiments were carried out to test how well ZP are able to distinguish between different subject groups depending on age and disease status. Linear Discriminant Analysis with leave-one-out scheme showed the accuracy, sensitivity and specificity of around 80%, suggesting good discrimination ability for both age and AMD. For comparison, classification based on the peak value and total amount of MP in a region with eccentricity of 4 degrees ([56]), features commonly used to assess MP, showed much lower accuracy (**S3 Table**; MANOVA: Wilk’s Lambda 0.899, p<0.0005).

Correlation of MP density with age has been a subject of a number of studies which showed different trends depending on the subject cohort and the method of MP measurement [14, 22, 26, 27, 54, 57, 58]. Any correlations that were shown (both positive and negative) were relatively weak and continue to be a subject of controversy. Results in this paper suggest that some age dependency may exist (**Table 3**, **Table 4**) with MP level tending to be higher in subjects under 50 (**Fig 4**).

A growing number of studies have been looking at distribution of MP in addition to its density [23, 24]. As subsequently postulated by Bernstein et al. [22]: “*most MPOD methods measure the density at the center of the retina relative to some eccentric location where macular pigment concentrations are assumed to be negligible*. *If an individual’s macular pigment is broadly distributed over many degrees*, *if it has an irregular distribution*, *or if supplementation increases lutein or zeaxanthin concentrations in the peripheral retina substantially*, *this assumption may not hold true*”. Most commonly, the findings related to MP distribution are presented in the form of plots of radial density profiles. Numerical indicators derived from the profiles typically include peak values and a rate of decrease in MP density as a function of eccentricity. The mean values, their variances and MP profile plots all tend to show significant individual variability. This was also the case in this study. However, MANOVA results presented here indicate that simple measures such as the peak and the gradient may not be capturing more subtle characteristics of MP distribution. As discussed in the “Analysis of spatial distribution patterns” section, rotational asymmetry and irregularity of the periphery may provide additional useful indicators. Quantification of these and further features is the subject of ongoing work, with the objective to provide more accurate means of studying MP distribution.

MP distribution has been examined in a variety of contexts, for example in investigations assessing the effects of supplementation [57, 59, 60], differences across various populations [14, 25, 61–66] or as a means of comparison of different MP measurement methods [5, 21, 33, 34, 56, 58, 67, 68]. Only a few studies examined MP distribution explicitly in the context of AMD. The most extensive was the Utah AREDS2 study [54] where MP was measured by dual-wavelength autofluorescence in subjects over 50 years of age. The main difference observed between AMD subjects and a normal population was the peakedness of MP distribution which was less pronounced in AMD subjects. Lower contrast between the central and peripheral MP density was observed across both subject groups. Irregular distributions were also observed but not explicitly apportioned to one of the groups. Results presented in this paper are in agreement with the AREDS2 findings (**Fig 4**).

Much work on the objective assessment of MP has as its goal identification of eyes at risk of AMD at the earliest possible stage. Whilst the mean results presented in this paper show promising trends, they do not provide evidence that the ZP representation of MRIA maps could be used to reliably assess *individual instances* of AMD. However, the increased accuracy of classification with a more comprehensive training set (cf PRNN and LOO classification experiments) suggests that with a larger cohort of subjects, especially those representing AMD-affected eyes, screening of at-risk individuals may become possible.

The quantification method employing ZP was applied here to MRIA maps, derived using a novel experimental method based on multispectral imaging and Gaussian Process Emulation. A natural question to ask is whether the results would be replicated for ZP representations of more conventional maps of MP distribution, such as, for example, autofluorescence.

**Fig 5:**
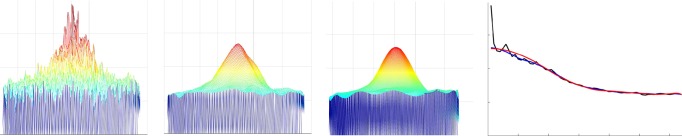
Fitting ZP to 2W-AF MP density maps. Left to right: 3D representation of a 2W-AF image; 3D representation of the image data after Gaussian smoothing (σ = 4); 3D representation of the ZP fit to the original 2W-AF image; and 2D profiles of the unprocessed 2W-AF data (black), Gaussian smoothed 2W-AF data (blue) and ZP fit (red).

To verify that the ability of ZP to represent MP distribution is not dependent on the imaging technique used, ZPs were fitted to thirteen images of MP density obtained with two-wavelength autofluorescence via a modified confocal scanning laser ophthalmoscope (Heidelberg HRA, Heidelberg, Germany) as described in Wüstemeyer et al. [69]. All images were from disease-free eyes and MP distribution was considered to be within normal limits. **Fig 5** shows comparison of 3D representations and profiles of (from left to right): 2W-AF original image data, the image data after averaging (Gaussian smoothing), ZP fit to the original 2W-AF data using the method described above, and radial profiles of the three above representations.

It is notable how closely ZP representations match the smoothed 2W-AF data in terms of both shape and magnitude. In particular the central peak observed in the original data loses its sharpness after averaging and attains a smoother profile. Trieschmann et al. [47] made a similar observation in their study comparing MP measurements with one- and two-wavelength autofluorescence methods. For the analysis of a single-wavelength fluorescence they used only averaged images, and for all experiments they avoided using the peak density “*because of the variability associated with a 1-pixel measurement*”.

This short supplementary experiment provides initial evidence that ZP representation can be effectively derived for a modality other than MRIA. FLIO topographic images of MPOD volume also show rounded profiles that are likely to be well represented by ZPs [20, 70]. Investigation of the full effectiveness of ZP representation for MP assessment in images originating from different imaging techniques would be a worthwhile project.

Overall, this small study has provided a proof of principle for the MP assessment method employing ZP. There is some evidence that MP levels are influenced by age and retinal health, and may also be related to dietary habits and nutritional supplementation [62, 64, 65]. Any larger classification study would have to take into account these and additional potentially contributing factors such as gender, smoking, body-mass index (BMI), diabetes and others.

## Conclusions

The results of this study suggest that Zernike polynomials (ZP) are capable of representing macular pigment (MP) distribution computed using the multispectral retinal image analysis (MRIA) technique, and potentially other imaging techniques such as 2W-AF and FLIO. Appropriately chosen subsets of Zernike basis functions were shown to characterise three aspects of MP distribution: magnitude, radial asymmetry and pattern irregularity. Using the ZP representation, MRIA MP maps could be classified according to both age and disease status with mean accuracy exceeding 80%.

These preliminary findings suggest that abstract descriptors such as ZPs may be capable of revealing visually non-obvious topographic features which can complement and enhance characterisation of spatial distribution.

## Supporting information

S1 Table The best tuneable classifier parameters chosen on the basis of classification performance on the entire dataset. The parameters in this table produced the best classification performance according to the evaluation by ROC analysis, and correspond to the performance indicators in S2 Table. KNN-k: the number of neighbours; KNN-c: the number of Zernike coefficients; SVM-c: the number of Zernike coefficients; SMNN-c: the number of Zernike coefficients; PRNN-c: the number of Zernike coefficients; PRNN-hn: the number of hidden neurons. Test groups are described in Table 1.
(DOCX)

S2 Table The best classification results for the four classifiers. The best classification results for the four classifiers using the meta-parameter settings shown in S1 Table. KNN-k: the number of neighbours; KNN-c: the number of Zernike coefficients; SVM-c: the number of Zernike coefficients; SMNN-c: the number of Zernike coefficients; PRNN-c: the number of Zernike coefficients; PRNN-hn: the number of hidden neurons. Acc.–accuracy; Sens.–sensitivity; Spec.–specificity. Test groups are described in Table 1.
(DOCX)

S3 Table Classification using LDA classifier with LOO selection on peak value and total amount of MP in a region with eccentricity of 4 degrees. Accuracy, sensitivity and specificity of classification according to the disease status regardless of age (1&2 vs 3), disease status in the age-matched groups (2 vs 3); age irrespective of disease status (1 vs 2&3); and age for subjects without AMD (1 vs 2). Test groups are described in Table 1.
(DOCX)
