# Supplementary material for: Investigating the potential of Zernike polynomials to characterise spatial distribution of macular pigment
Source: PLoS One. 2019 May 24;14(5):e0217265. doi: 10.1371/journal.pone.0217265 (PMC6534297; doi:10.1371/journal.pone.0217265)
Supplement: S1 Table — The parameters in this table produced the best classification performance according to the evaluation by ROC analysis, and correspond to the performance indicators in S2 Table. KNN-k: the number of neighbours; KNN-c: the number of Zernike coefficients; SVM-c: the number of Zernike coefficients; SMNN-c: the number of Zernike coefficients; PRNN-c: the number of Zernike coefficients; PRNN-hn: the number of hidden neurons. Test groups are described in Table 1. (DOCX) [file pone.0217265.s001.docx]

**Table S1. The best tuneable classifier parameters chosen on the basis of classification performance on the entire dataset.**

| Test groups |  | Classifier | | | | | |
| --- | --- | --- | --- | --- | --- | --- | --- |
|  |  | KNN-k | KNN-c | SVM-c | SMNN-c | PRNN-c | PRNN-hn |
| Not- centred |  |  |  |  |  |  |  |
| 1&2 vs 3 |  | 1 | 15 | 80 | 105 | 60 | 416 |
| 2 vs 3 |  | 1 | 15 | 105 | 105 | 105 | 424 |
| 1 vs 2&3 |  | 9 | 15 | 60 | 15 | 15 | 92 |
| Centred |  |  |  |  |  |  |  |
| 1&2 vs 3 |  | 1 | 15 | 105 | 105 | 15 | 373 |
| 2 vs 3 |  | 1 | 15 | 80 | 80 | 15 | 414 |
| 1 vs 2 3 |  | 1 | 15 | 80 | 80 | 15 | 414 |

The parameters in this table produced the best classification performance according to the evaluation by ROC analysis, and correspond to the performance indicators in Table S2. KNN-k: the number of neighbours; KNN-c: the number of Zernike coefficients; SVM-c: the number of Zernike coefficients; SMNN-c: the number of Zernike coefficients; PRNN-c: the number of Zernike coefficients; PRNN-hn: the number of hidden neurons. Test groups are described in Table 1.
