# Supplementary material for: Investigating the potential of Zernike polynomials to characterise spatial distribution of macular pigment
Source: PLoS One. 2019 May 24;14(5):e0217265. doi: 10.1371/journal.pone.0217265 (PMC6534297; doi:10.1371/journal.pone.0217265)
Supplement: S2 Table — The best classification results for the four classifiers using the meta-parameter settings shown in S1 Table. KNN-k: the number of neighbours; KNN-c: the number of Zernike coefficients; SVM-c: the number of Zernike coefficients; SMNN-c: the number of Zernike coefficients; PRNN-c: the number of Zernike coefficients; PRNN-hn: the number of hidden neurons. Acc.–accuracy; Sens.–sensitivity; Spec.–specificity. Test groups are described in Table 1. (DOCX) [file pone.0217265.s002.docx]

**Table S2. The best classification results for the four classifiers.**

| Test groups |  | Classifier | | | | | | | | | | | |
| --- | --- | --- | --- | --- | --- | --- | --- | --- | --- | --- | --- | --- | --- |
|  |  | KNN | | | SVM | | | SMNN | | | PRNN | | |
| Not-centred |  | Acc. | Sens. | Spec. | Acc. | Sens. | Spec. | Acc. | Sens. | Spec. | Acc. | Sens. | Spec. |
| 1&2 vs 3 |  | 0.77 | 0.89 | 0.43 | 0.80 | 0.94 | 0.39 | 0.72 | 0.80 | 0.48 | 0.86 | 0.89 | 0.63 |
| 2 vs 3 |  | 0.65 | 0.71 | 0.58 | 0.71 | 0.68 | 0.74 | 0.71 | 0.68 | 0.74 | 0.81 | 0.85 | 0.77 |
| 1 vs 2&3 |  | 0.77 | 0.68 | 0.84 | 0.68 | 0.60 | 0.74 | 0.73 | 0.64 | 0.80 | 0.88 | 0.74 | 0.96 |
| Centred |  |  |  |  |  |  |  |  |  |  |  |  |  |
| 1&2 vs 3 |  | 0.73 | 0.80 | 0.52 | 0.75 | 0.87 | 0.42 | 0.75 | 0.86 | 0.42 | 0.86 | 0.95 | 0.36 |
| 2 vs 3 |  | 0.71 | 0.68 | 0.74 | 0.62 | 0.72 | 0.52 | 0.67 | 0.74 | 0.58 | 0.83 | 0.95 | 0.73 |
| 1 vs 2&3 |  | 0.66 | 0.68 | 0.64 | 0.71 | 0.62 | 0.78 | 0.75 | 0.70 | 0.78 | 0.90 | 0.90 | 0.90 |

The best classification results for the four classifiers using the meta-parameter settings shown in Table S1. KNN-k: the number of neighbours; KNN-c: the number of Zernike coefficients; SVM-c: the number of Zernike coefficients; SMNN-c: the number of Zernike coefficients; PRNN-c: the number of Zernike coefficients; PRNN-hn: the number of hidden neurons. Acc. – accuracy; Sens. – sensitivity; Spec. – specificity. Test groups are described in Table 1.
