# Supplementary material for: Investigating the potential of Zernike polynomials to characterise spatial distribution of macular pigment
Source: PLoS One. 2019 May 24;14(5):e0217265. doi: 10.1371/journal.pone.0217265 (PMC6534297; doi:10.1371/journal.pone.0217265)
Supplement: S3 Table — Accuracy, sensitivity and specificity of classification according to the disease status regardless of age (1&2 vs 3), disease status in the age-matched groups (2 vs 3); age irrespective of disease status (1 vs 2&3); and age for subjects without AMD (1 vs 2). Test groups are described in Table 1. (DOCX) [file pone.0217265.s003.docx]

**Table S3. Classification using LDA classifier with LOO selection on peak value and total amount of MP in a region with eccentricity of 4 degrees.**

| Test groups |  | All coefficients | | |  | |
| --- | --- | --- | --- | --- | --- | --- |
| Not-centred |  | Accuracy | Sensitivity | Specificity | |  |
| 1&2 vs 3 |  | 0.57 | 0.55 | 0.58 | |  |
| 2 vs 3 |  | 0.60 | 0.71 | 0.45 | |  |
| 1 vs 2&3 |  | 0.58 | 0.55 | 0.62 | |  |
| 1 vs 2 |  | 0.63 | 0.62 | 0.64 | |  |

Accuracy, sensitivity and specificity of classification according to the disease status regardless of age (1&2 vs 3), disease status in the age-matched groups (2 vs 3); age irrespective of disease status (1 vs 2&3); and age for subjects without AMD (1 vs 2). Test groups are described in Table 1.
